# Complexity of Central Processing in Simple and Choice Multilimb Reaction-Time Tasks

**DOI:** 10.1371/journal.pone.0090457

**Published:** 2014-02-28

**Authors:** Matthieu P. Boisgontier, George F. Wittenberg, Hakuei Fujiyama, Oron Levin, Stephan P. Swinnen

**Affiliations:** 1 Movement Control and Neuroplasticity Research Group, Group Biomedical Sciences, KU Leuven, Leuven, Belgium; 2 Geriatric Research, Education and Clinical Center, VA Maryland Health Care System, Baltimore, Maryland, United States of America; 3 Department of Neurology, University of Maryland School of Medicine, Baltimore, Maryland, United States of America; 4 Leuven Research Institute for Neuroscience & Disease (LIND), Leuven, Belgium; Centre national de la recherche scientifique, France

## Abstract

The default mode of the motor system is a coupling between limbs. However, in some movements, a decoupling is required and thus calls for selection and facilitation/inhibition processes. Here, we investigate the relative contribution of recruitment versus selection processes to the overall processing complexity. To this aim we proposed a new multilimb reaction-time task (MUL-RT). Simple, choice and normalized (choice minus simple) RT were analysed together with error rates in thirty-six young adults for 15 coordination modes including all possible configuration of limb recruitment. Simple and normalized RTs were respectively assumed to be indicative of the recruitment and selection processes. Results supported a model of coupling/decoupling interactions respectively reporting weak, intermediate and strong interaction for selecting diagonal, ipsilateral and homologous limbs. Movement laterality (left vs. right) had no effect on selection complexity, whereas selecting upper limbs was less challenging than selecting lower limbs. Results in the different coordination modes suggested that recruitment complexity decreased as follows: 3 limbs = 4 limbs>2 limbs (homologous, ipsilateral and diagonal)>1 limb, and selection complexity as follows: 2 diagonal limbs>3 limbs>2 ipsilateral limbs>1 limb = 2 homologous limbs>4 limbs. Based on these ordinal scales of recruitment and selection complexity, we extrapolated the overall processing complexity of the simple and choice MUL-RT. This method was efficient in reproducing the absolute results we obtained on a ratio scale (ms) and demonstrated that processing complexity in simple RT was mainly governed by the ‘recruitment principle’ (the more limbs recruited the lower the performance), whereas contributions of recruitment and ‘selection principle’ (nature of the coordination determines performance) to overall processing complexity were similar in choice RT.

## Introduction

Reaction time (RT) refers to the time elapsing between a stimulus and a detectable movement, a physical change, or an action, occasioned by the occurrence of the stimulus [1]. RT is traditionally described by a Stimulus-Processing-Response framework whereby the brain’s processing capacity mediates the relationship between the stimulus and the response, including stimulus identification, appropriate response selection, and response programming [2]. In psychometric psychology, RT is therefore considered to be an index of speed and efficiency of central processing afforded by the brain [3]. Three main types of RT can be differentiated. *Simple* RT tasks require the participant to respond to the presence of a single stimulus. *Recognition* RT tasks require the participant to respond when one specific stimulus appears and to withhold his response when other types of stimuli are presented. *Choice* RT tasks require distinct responses for each type of stimulus. Simple RT is shorter than a recognition RT, and choice RT is longest of all [4]. Longer RT is assumed to be indicative of more complex processing requirements and/or the degree of integrity of the central nervous system. Although cognitive aspects associated with RT have been studied intensively, only limited attention has been allocated to the role of motor-related parameters [5]. More specifically, few studies have addressed whether the particular combination of limbs affects task performance.

Here, we first investigate the levels of coupling and decoupling between limbs at the stage of selection processes. Second, we posit that processing complexity associated with a motor task is determined by a weighted combination of both (1) the number of limbs to be involved in the movement (i.e., recruitment principle) and (2) the coupling/decoupling interactions involved in a given coordination mode (i.e., selection principle).

### Coupling and Decoupling of Effector-specific Brain Networks

#### The brain as a functional space

Brain crosstalk can primarily occur either between interconnected control centres distributed across the hemispheres (interhemispheric) or between neuronal populations within lateralized control regions (intrahemispheric) [6]. In the context of motor control, each limb is associated with a specific sensorimotor network consisting of primary motor, primary sensory, premotor and supplementary motor areas [7]. In the 1970s, Kinsbourne and Hicks [8], [9] proposed consideration of the brain as a functional space wherein the functional distance between any two cerebral areas decreases with the extent to which they collaborate (facilitate) or compete (interfere) with each other for concordant and discordant movements, respectively. This model suggested that brain areas within a hemisphere (ipsilateral) are functionally closer than interhemispheric brain areas with the exception of callosal (interhemispheric) homologous areas representing the closest connections. As such, functional distance does not necessarily comply with physical distance.

**Figure 1:**
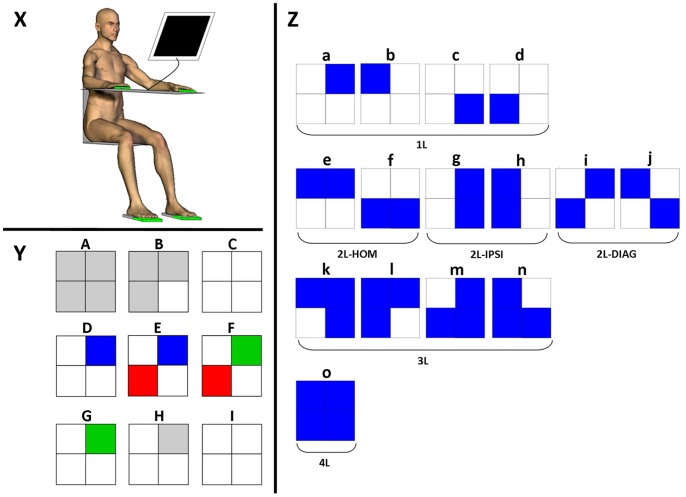
Study setup. X. Participant setup. Participants were seated in front of a PC-screen, their forearms resting on a table and their fingers and forefeet on tablets with capacitive proximity switches (in green). **Y. Instance of a trial sequence represented on the PC-screen.** The right and left upper squares represent the right and left hands whereas the right and left lower squares represent the right and left feet, respectively. (A) Squares are grey when limbs are not in contact with the tablets. (B) They turn white as soon as a limb contacts the corresponding tablet. (C) A trial starts as soon as all limbs are in contact with the tablets. (D) When a square turns blue, this is the stimulus for the participant to release contact with the corresponding tablet as quickly as possible. (E) If the participant lifts the incorrect limb(s), the corresponding square(s) turn(s) red. (F) If he lifts the correct limb(s), the corresponding square(s) turn(s) green. (G) A trial is not validated until the response is fully correct, i.e., without any red square on the screen. (H) As soon as the trial is validated, the green squares turn back to grey. (I) Participants have to reposition all limb segments on the tablets to start a new trial. **Z.** **Coordination modes and clusters.** The 15 possible coordination modes (‘a’ to ‘o’) were grouped according to 5 clusters (1L, 2L-HOM, 2L-IPSI, 2L-DIAG, 3L, 4L) based on the number of limbs to be recruited (1, 2, 3 or 4) and the coupling/decoupling interactions involved. (L = limb; DIAG = diagonal; IPSI = ipsilateral; HOM = homologous).

The functional proximity of homologous areas is supported by the occurrence, in children, of mirror movements during intended unilateral movement of the opposite limb [10], [11]. In adults, while overt mirror movements are rare, homologous spread of motor neuronal activity may be demonstrated. Behavioral studies have revealed this spread when movements with different amplitudes [12], [13], [14], [15] and/or directions [16], [17], [18] were performed simultaneously. In these studies, an assimilation effect emerged with amplitudes and directions tending to become similar to each other. Electromyographic studies have shown that, when moving a single limb, neural spread is more pronounced in the homologous relative to the ipsilateral and diagonal muscles [19], [20] (Please see Figure 1Z, 2L panel, for an illustration of the 2-limb configurations). Studies using transcranial magnetic stimulation (TMS) of the motor cortex have demonstrated that responses evoked in a limb are facilitated by the contraction of the homologous muscles of the opposite limb [21], [22], [23]. Results of interlimb coordination studies have also supported the functional proximity of homologous brain areas. In these studies, seated healthy young [24], [25], [26] and older adults [27] performed cyclical flexion/extension movements of 2 limbs in the same (in phase) or opposite direction (antiphase) according to three conditions: ipsilateral, homologous and diagonal. Results revealed that performance in terms of accuracy (absolute error of the relative phase), variability (of cycle duration) and amplitude was better in the homologous relative to the ipsilateral and diagonal conditions. Nerve stimulation [28] and TMS studies [29], [30], [31] have also supported the functional connection between ipsilateral areas as they demonstrated that contraction of one limb facilitated movement of the ipsilateral limb. This facilitation seemed to result from disinhibition [30], [31].

The corpus callosum connects interhemispheric areas with a greater proportion of homologous versus heterologous regions [32] and thereby provides an anatomical substrate for the relative difference between homologous and diagonal limbs in terms of functional distance. Conversely, the anatomical substrate linking ipsilateral areas or subserving the ipsilateral limbs is less clear. Indirect pathways involving extracortical structures or even spinal connections may subserve these ipsilateral interactions, as well as the aforementioned homologous and diagonal interactions [33].

#### A model of coupling/decoupling interactions

**Figure 2:**
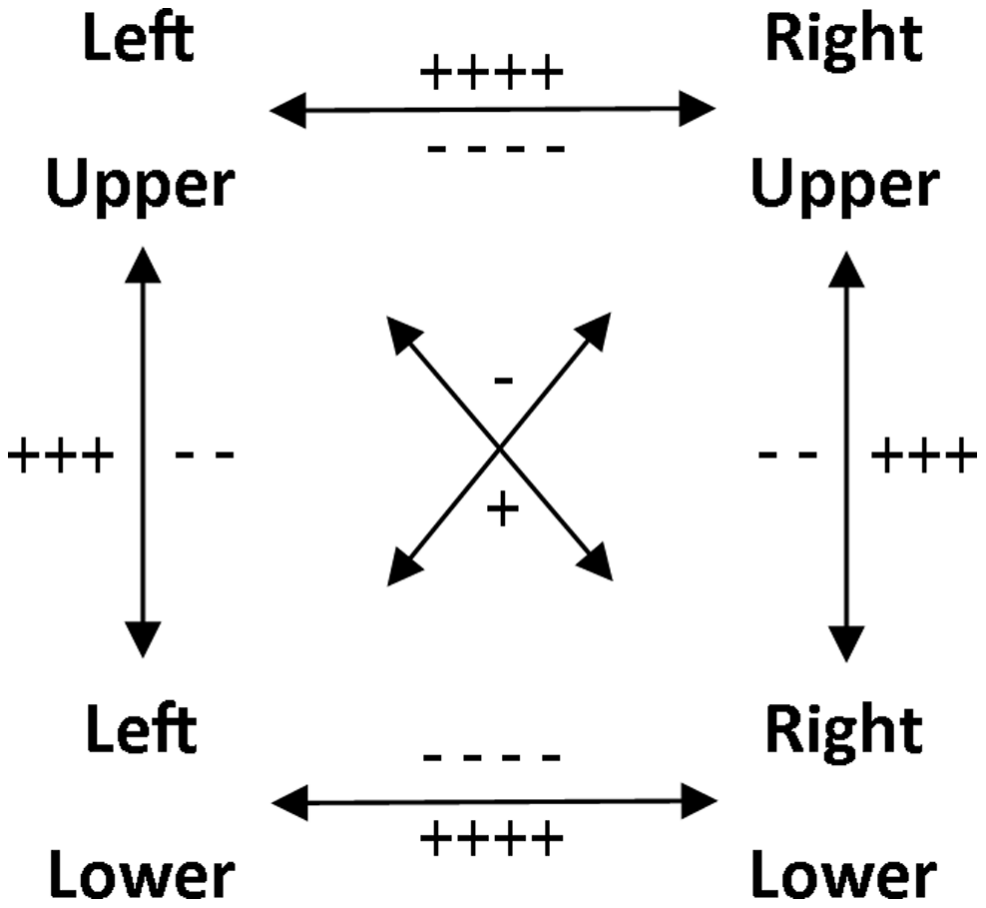
The model of coupling/decoupling interactions. The quantity of ‘+’ and ‘–’ signs respectively convey the strength of the coupling and decoupling interactions, e.g., ‘++++’ is a very strong coupling whereas ‘−’ is a very weak decoupling.

In sum, at first glance, the functional space model is potentially appropriate to account for behavioral performance differences in movements requiring various limb combinations. However, studies testing the four limbs in 4-choice/1-limb RT tasks [34], [35], [36], [37] with congruent mapping of the stimuli relative to the effectors (Figure 1Z, 1L panel) have consistently contradicted the functional space model. In these studies, participants were typically presented four different stimuli with each stimulus being uniquely associated with one of the four limbs. Participants were instructed to respond as quickly and as accurately as possible with one limb according to the presented stimulus. Results revealed that, with reference to the correct limb, errors (i.e., moving a limb different from that indicated by the stimulus) more commonly appeared on the ipsilateral (50 to 98% of the total number of errors) relative to the homologous (0 to 36%) and diagonal limbs (0 to 18%) [34], [35], [36], [37]. Following the functional space model, these results would suggest that homologous areas are functionally quite distant from each other (weak interference), which is unlikely given that there is known robust anatomical and functional connection between these areas through the corpus callosum [32], [38]. The reason for this inconsistency may be that the original functional distance concept does not differentiate between coupling (activation) and decoupling (inhibition) interactions. Assuming weak inhibitory together with strong excitatory interactions between ipsilateral areas (Figure 2) can account for the difficulty in preventing/suppressing ipsilateral errors in 4-choice/1-limb RT tasks. Alternatively, the scarceness of homologous errors could be explained by the ability to overcome the excitatory interaction by recruitment of interhemispheric inhibitory pathways (Figure 2).

Limbs tend to coordinate automatically toward a natural coordination mode (in phase or antiphase) and tasks that deviate from this intrinsic mode are challenging [39], [40], [41], [42]. The default mode of the motor system is therefore a basic coupling between the limbs. However, in other movements, a decoupling is required and thus calls for inhibitory interactions. In addition to results in 4-choice/1-limb RT studies [32], [33], [34], [35], the strong inhibitory interaction between homologous areas is also supported in children by the decreasing prevalence and intensity of mirror movements with age [10], [11] as these decreases coincide with the increasing maturity of the inhibitory system [43]. Furthermore, in performing daily activities, we are frequently involved in selecting either the dominant or non-dominant hand to reach for objects, whereas selecting between the upper and lower limb segments is much less prominent because the functions they afford are more distinct. This suggests that single upper limb selection and thus deselection of the contralateral limb are highly optimized processes that have evolved from an intrinsically bimanual default state.

Even though the corpus callosum is thought to play a major role in coupling and decoupling homologous and, to a weaker degree, diagonal movements [32], [33], [44], [45], inhibitory interactions are also likely to occur between intrahemispheric areas. Indeed, TMS studies using the electromyographic silent period as an indicator of cortical inhibition in cyclical movements have shown that the level of inhibition of cortical motor pathways is higher between ipsilateral, relative to diagonal limbs [46], [47]. Yet, no study compared relative levels of inhibition between the three 2-limb patterns so far (homologous, ipsilateral, and diagonal). In addition to results in 4-choice/1-limb RT studies [34], [35], [36], [37], the weakness of inhibitory interactions between ipsilateral relative to homologous areas is also supported by results in 2-limb coordination studies [26], [27]. In these studies, the anti-directional (antiphase) mode is produced with significantly less accuracy than the isodirectional (in phase) mode during ipsilateral coordination, whereas this is less the case for the homologous limb combinations.

In summary, the brain is considered as a highly interconnected neural space with different gradients of coupling and decoupling interactions. Activation in one area may therefore spread to other areas to different extents. The latter requires recruitment of inhibitory mechanisms to enable selective movement generation. The model of coupling/decoupling interactions we propose here (Figure 2) incorporates Kinsbourne and Hicks’ concept of functional distance but with the important addition to differentiate between excitatory and inhibitory interactions. Indeed, coupling and decoupling interactions are stronger (neural distance is shorter) between homologous areas relative to ipsilateral areas with the diagonal ones being the weakest (most distant). This revised model could be considered as a potential tool for explaining how the *selection principle* (please see below) determines the complexity of the central processing for controlling coordinated movements.

### Determinants of Motor Control Complexity: Recruitment and Selection Principles

Here, we posit that overall processing complexity of a task might be determined by the recruitment (the more effector-specific networks recruited the lower the performance) and selection (the coupling/decoupling interactions involved in a given coordination mode determine performance) principles. The possibility for the recruitment principle to account for the complexity of central processing of coordinated movements has been investigated by Swinnen and collaborators [48]. This study tested simple RTs in 1-, 2-homologous-, 3-, and 4-limb conditions. Results showed no significant difference between the 1-limb and 2-homologous-limb conditions but both these conditions demonstrated shorter simple RTs than the 3-limb and 4-limb conditions. This result suggested a prominent role of the recruitment principle in simple RTs. However, adding the two other 2-limb conditions (ipsilateral and diagonal) to the experimental design would give more information about the validity of simple RTs as a measure of recruitment complexity. As demonstrated in the previous section, these three conditions carry different levels of selection complexity. Therefore, if simple RT is a valid measure of recruitment complexity, one would expect similar simple RTs in the three 2-limb conditions. As shown in the previous section, between-limb interactions have been studied quite intensively. However, the focus has primarily been on interactions within limb couples. Furthermore, the recruitment and selection principles have never been tested together. How these interact with each other and whether they are sufficient to account for results in all possible limb combinations has never been investigated. Yet, this information would provide new insights into the mechanisms that govern the level of complexity of multilimb motor control.

Here, we propose a new and complete paradigm testing 15 coordination modes including all possible configurations of limb recruitment in simple and choice RT conditions. Based on this paradigm we first analyse inhibitory and excitatory interactions governing the selection of effector-specific brain networks to test our model of coupling/decoupling interactions. Second, we investigate for the first time the relative impact of the determinants of processing complexity for controlling movement. Specifically, we hypothesize that processing complexity for controlling movement in simple RTs is mainly determined by the recruitment principle whereas choice RTs are driven by a combination of the recruitment and selection principles.

## Materials and Methods

### Participants

Thirty six young adults (mean age, 22 years; range, 19–28 years; 15 females) were recruited to participate in the study. All participants were right-handed and right-footed [49]. At the time of testing, participants reported having no neuromuscular impairment. All participants gave written informed consent before the experiment. Experimental procedures were approved by the Ethics Committee of Biomedical Research at the KU Leuven in accordance with the Code of Ethics laid down by the World Medical Association (Declaration of Helsinki).

### Setup

Participants were seated in front of a PC-screen (distance approx. 0.5 m), their forearms resting on a table and their fingers and forefeet on tablets with capacitive proximity switches (Pepperl Fuchs CBN5-F46-E2, sampling frequency: 1000 Hz) (Figure 1X). Four squares representing the four limb segments were presented on the PC-screen. Mapping of the stimuli was maximally congruent relative to the effectors. When all 4 limb segments contacted the tablets, some of the squares turned blue after a randomly varying time ranging from 2 to 4 s (Figure 1Y). In response to this stimulus, participants had to release contact with the corresponding tablets as quickly and as correctly as possible by lifting the indicated limb segment(s). Fifteen limb segment conditions referred to as ‘coordination modes’ were tested (Figure 1Z). This 15-condition/4-limb task is referred to as the multilimb reaction-time task (‘The MUL-RT’) including two variants depending on whether a simple (Simple MUL-RT) or a choice RT (Choice MUL-RT) paradigm is tested.

### Procedures

**Figure 3:**
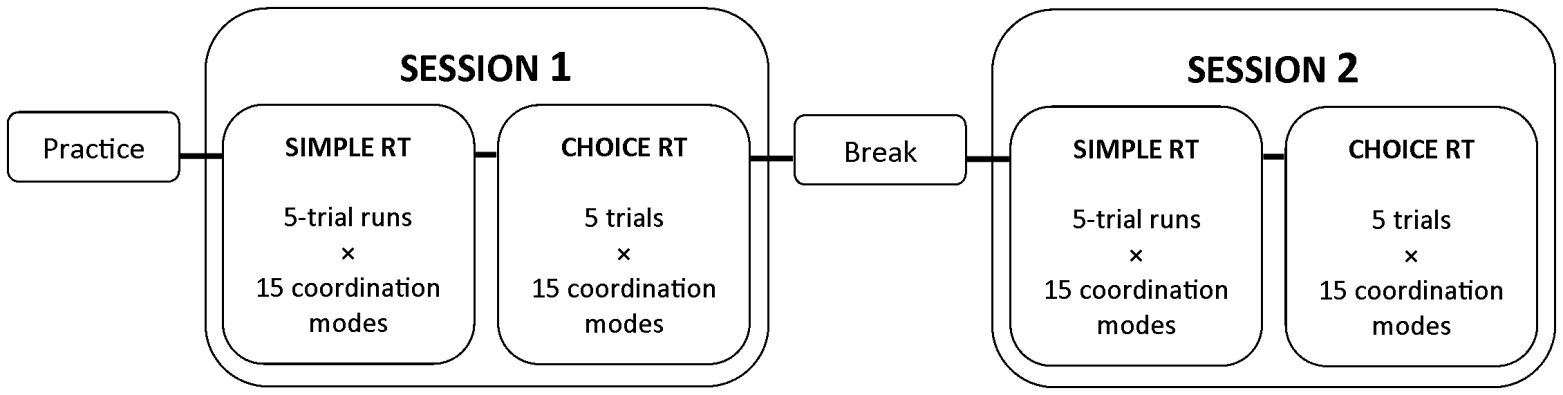
Study design. The simple MUL-RT and choice MUL-RT blocks were composed of 5-trial runs×15 coordination modes and 5 trials×15 coordination modes, respectively. Conditions were randomly distributed within both blocks.

For familiarization purposes, participants were instructed to perform each coordination mode prior to initiation of the experiment. The experimental design was composed of two sessions divided by a 5-min break (Figure 3). The two sessions consisted of two identical blocks each and were used to inquire about possible between-session practice and/or fatigue effects as well as to test the robustness of the results against practice effects. The first block of each session was composed of randomized 5-trial runs of each of the 15 coordination modes. Before each run, the participant viewed a printed copy of the figure that would appear on the screen for the subsequent 5-trial run (predictive − no choice required). This first block of each session was called ‘simple RT’. The second block consisted of performing 75 trials (5 trials×15 coordination modes) in randomized order (non-predictive − choice required). This second block was called ‘choice RT’. In total, each participant performed 300 trials.

### Data and Statistical Analysis

For error-free trials, the time interval between the onset of the visual stimulus and the time the participant performed the correct coordination mode (i.e., when released limbs corresponded to the screen stimulus) were averaged for each of the 15 coordination modes in the simple and choice RT blocks. The number of errors (i.e., when participants released an incorrect limb) was averaged across all trials of a given condition and then multiplied by 100 to be expressed as a percentage. Time and error data were normalized by subtracting the absolute measures of the simple RT condition from the choice RT condition. This normalization procedure was intended to selectively focus on the processing component of the Stimulus-Processing-Response paradigm and more specifically on the selection-related processing. Coordination modes were arranged on the basis of the number of effector-specific networks involved [7] and the coupling/decoupling interaction they conveyed. This arrangement resulted in six coordination clusters composed of (Figure 1Z): four 1-limb coordination modes (a, b, c, d), two 2-homologous-upper/lower-limb coordination modes (e, f), two 2-ipsilateral-right/left-limb coordination modes (g, h), two 2-diagonal-limb coordination modes (i, j), four 3-limb coordination modes (k, l, m, n), and one 4-limb coordination mode (o). These six coordination clusters were respectively called: 1L, 2L-HOM, 2L-IPSI, 2L-DIAG, 3L and 4L.

### Coupling and Decoupling Interactions

#### Activation

Because an excitatory interaction is assumed to facilitate activation, greater excitatory interaction within an effector-specific network couple will improve RT performance. Therefore, the normalized RT duration required to move two limbs was considered to be indicative of the level of excitatory interaction (i.e., coupling). As we aimed at investigating each single excitatory interaction between limbs independently, we only compared normalized times of the three 2-limb clusters. The excitatory component of our model of coupling/decoupling interactions (illustrated by “+” signs in Figure 2) was tested by means of a 1-way analysis of variance (ANOVA) with the factor 2 Limbs (3 levels: 2L-HOM, 2L-IPSI, 2L-DIAG).

### Inhibition

Error rate was considered to be indicative of the failure of the inhibitory interaction between limbs. Therefore, to test the inhibitory component of our model (i.e., decoupling) (illustrated by “−” signs in Figure 2), error rates were analysed by means of a 1-way ANOVA with the factor Coordination Cluster (5 levels: 1L, 2L-HOM, 2L-IPSI, 2L-DIAG, 3L). In this ANOVA, errors within level 1L reflected failure of either homologous, ipsilateral or diagonal inhibition, 2L-HOM failure of ipsilateral+diagonal inhibition, 2L-IPSI failure of homologous+diagonal inhibition, 2L-DIAG failure in homologous+ipsilateral inhibition, and 3L failure of homologous+ipsilateral+diagonal inhibition. To make the error-rate comparisons meaningful among conditions involving a different number of potential locations of error, normalized error rates in conditions involving 1, 2 and 3 limbs were respectively divided by 3, 2 and 1 (adjusted error).

To identify how errors were distributed in the 1L condition, adjusted error rates were analysed by means of a 1-way ANOVA with the factor 1 Limb (3 levels: Homologous failure, Ipsilateral failure, Diagonal failure).

#### Processing complexity for the control of limb movements

For *simple RTs*, the level of complexity was assumed to be low on stimulus identification and effector selection because there was only a single stimulus configuration to identify. As this stimulus configuration was precued, selecting the appropriate limb(s) could be completed in advance. Thus, complexity resided in recruitment of the appropriate network and generation of the response involving 1, 2, 3 or 4 limbs following the stimulus display. Therefore, performance on simple RTs was considered to be indicative of the number-related complexity.

For choice RTs, limb selection and response programming occurred under time pressure and took place in a more complex context compared to simple RTs with prevalent coordination constraints requiring more pronounced inhibitory recruitment for deselection of some limbs in the presence of more salient selection of others. Therefore, *normalized data* (choice minus simple RT) were considered to be indicative of the selection complexity.

### Recruitment and Selection Complexity Associated with Upper vs. Lower and Right vs. Left Limbs

To test the effect of limb and body side on processing complexity for controlling movement, data were clustered by limb irrespective of the coordination mode. To analyse recruitment complexity, *simple RTs* were analysed by means of a 2×2×2 full repeated ANOVA with the factors Session (2 levels: Session 1, Session 2), Limb (2 levels: Upper limbs, Lower limbs) and Laterality (2 levels: Right limbs, Left limbs). As a very low error rate was expected in the simple RT condition, this variable was not analysed here.

To analyse selection complexity, *normalized RT* and *normalized error-rate* data were analysed by means of 2×2×2 (Session×Limb×Laterality) full repeated measures analyses of variance (ANOVAs).

### Recruitment Complexity of Coordination Clusters

To test complexity associated with the number of effector-specific networks to be recruited, *simple RTs* were analysed by means of a 2×5 full-repeated ANOVA with the factors Session and Coordination Cluster (6 levels: 1L, 2L-HOM, 2L-IPSI, 2L-DIAG, 3L, 4L). As a very low error rate was expected in the simple RT condition, this variable was not analysed here.

### Selection Complexity of Coordination Clusters

To test complexity associated with the selection of a given coordination mode, *normalized RT* measures were submitted to a 2×6 full repeated measures ANOVA (Session × Coordination Clusters). Coordination cluster 4L would necessarily result in an absence of error as all limbs had to be recruited. Therefore, normalized error rates were analysed by means of a 2×5 (Session × Coordination Cluster) ANOVA with repeated measures on both factors.

### Weighting of Recruitment and Selection Complexity

From previous analyses we aimed at determining ordinal scales of complexity associated with the number and selection principles. To demonstrate that the relative contribution/weighting of these two scales can determine overall complexity in both simple and choice RT conditions, we intended to match the ordinal level of overall complexity based on this relative weighting and the actual (ratio) performance in single and choice MUL-RT. *Absolute RTs* were analysed by means of a 2×2×6 full-repeated ANOVA with the factors Session, Block (2 levels: Simple RT, Choice RT) and Coordination Cluster.

For all statistical analyses, the level of significance was set at *p*<0.05, 2-sided. P-values of ANOVAs were corrected for sphericity (*corr. p*) using the Greenhouse-Geisser method when Mauchly’s test was significant. To perform ANOVAs, error-rate data were transformed using the square root transformation [50]. When ANOVAs revealed significant effects, post-hoc tests (Tukey HSD, which corrects for multiple comparisons) were conducted to identify the loci of these effects. Main effects or interactions were not reported when a higher-order interaction reached significance [51]. Partial eta squared (η^2^_P_) were reported to indicate small (≤0.01), medium (≤0.06) and large (≤0.14) effect sizes [52].

## Results

**Figure 4:**
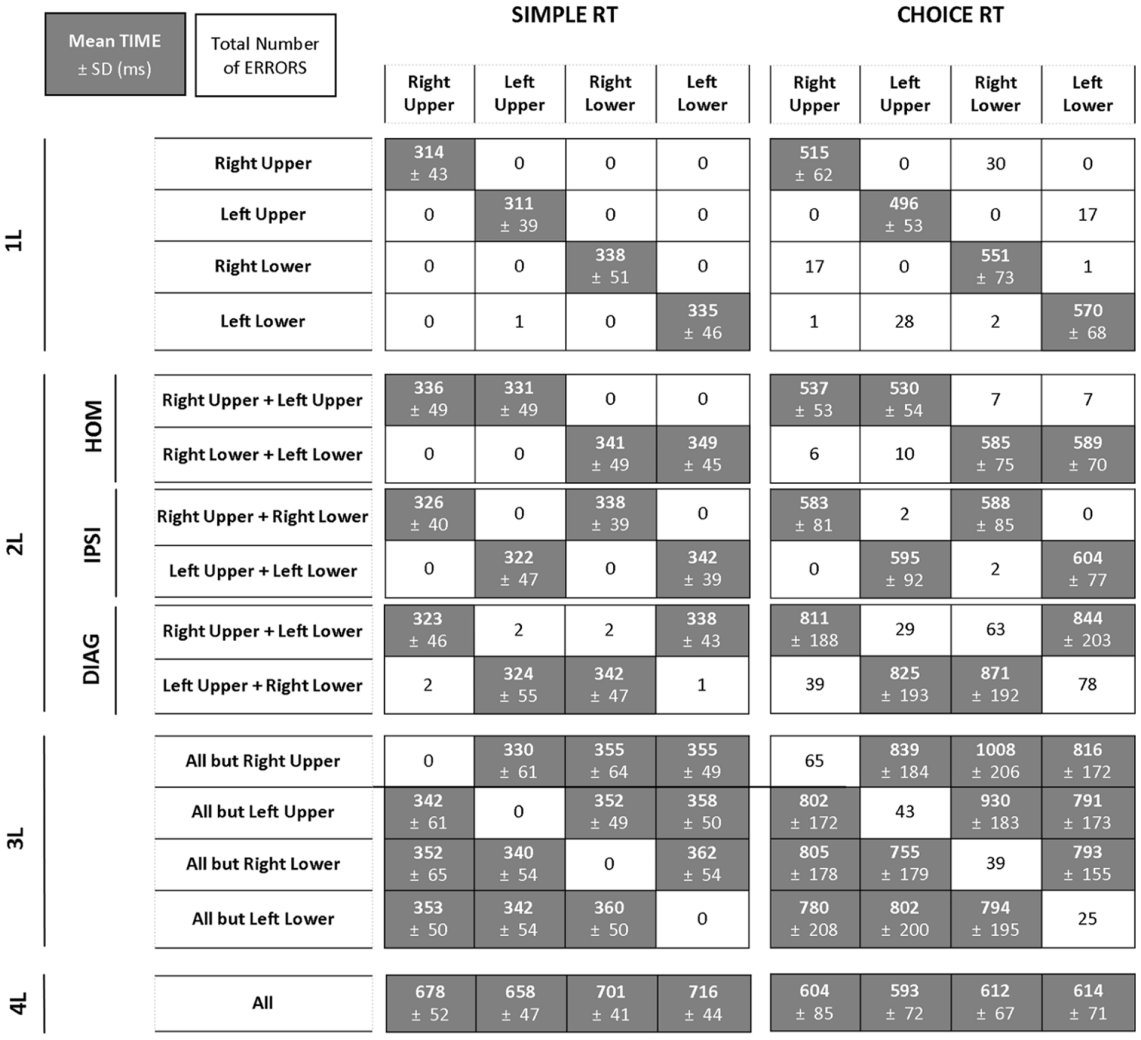
Descriptive results. Mean time ± SD (white text, grey fill) and total number of errors (black text, white fill) for each limb in the simple RT and choice RT conditions as a function of coordination modes. The mean time was computed over free-of-error trials. For a given trial the error was assigned to the first limb that released contact when it should not have. (L = limb; DIAG = diagonal; IPSI = ipsilateral; HOM = homologous).

Descriptive results of reaction time and error rate (Mean ± SD) are reported in Figure 4. As expected, very few errors were observed in the simple RT condition with a total of 8 errors out of 10800 trials. The total number of errors in the choice RT condition was 511 (4.7%).

### Coupling and Decoupling Interactions

Here, we report results that aim at testing the validity of our model of coupling/decoupling interactions. In addition these results provide a basis to explain differences of *selection* complexity among different coordination patterns.

#### Activation

**Figure 5:**
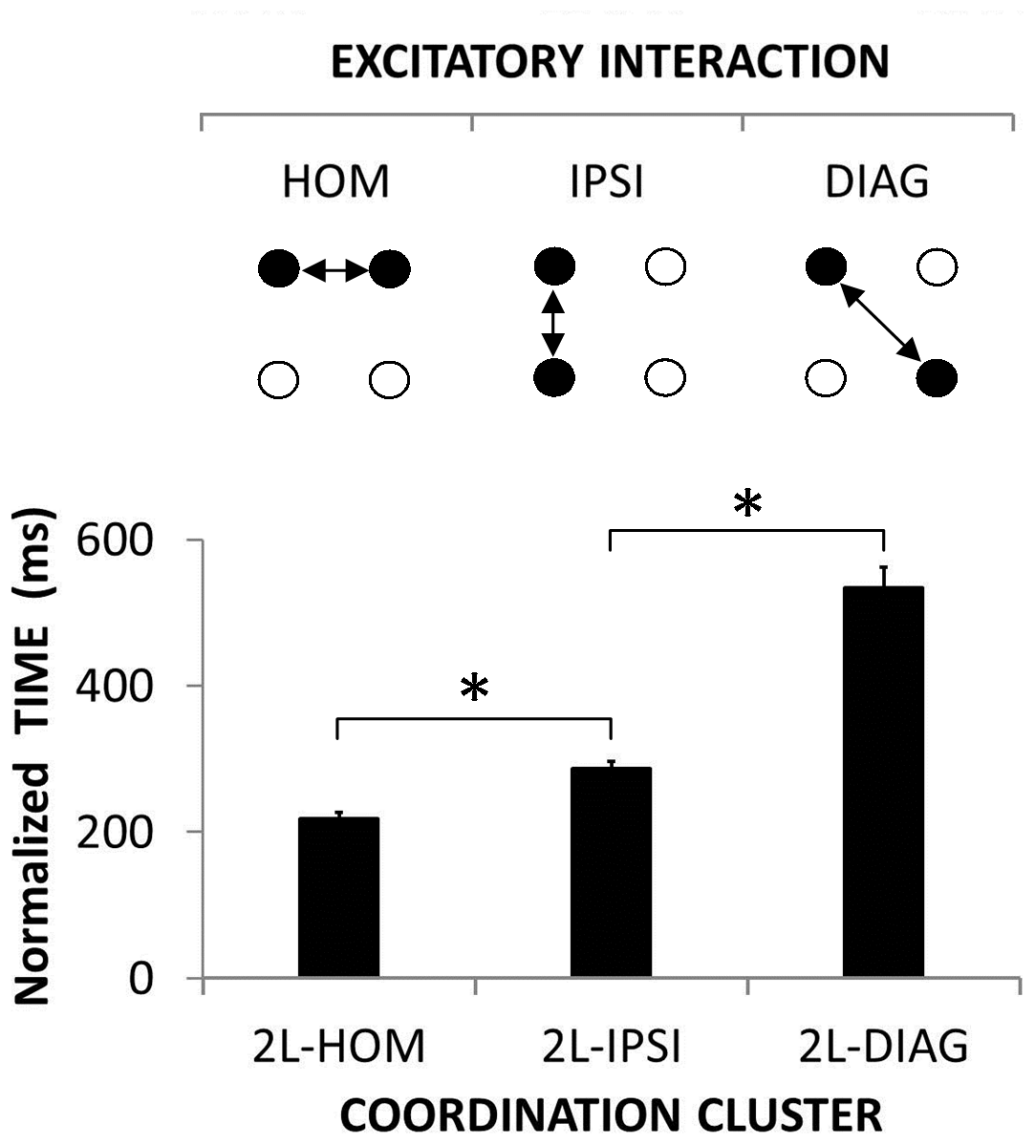
Coupling of effector-specific networks. Normalized time as a function of the three 2-limb coordination clusters. Normalized time reflects excitatory interaction, i.e., the shorter the RT the stronger the interaction. Each circle stands for one effector-specific brain network. Black-filled circles illustrate networks associated with moving effectors and white-filled circles represent non-moving effectors. Arrows depict excitatory interaction between moving effectors. (Mean ± SEM; L = limb; DIAG = diagonal; IPSI = ipsilateral; HOM = homologous; * = significant difference).

For *normalized RTs*, the 1-way (2 Limbs) ANOVA demonstrated a significant main effect [F(2, 70) = 150.35; *corr. p*<0.001; η^2^_P_ = 0.811] (Figure 5). Post-hoc tests revealed that normalized RTs in the 2L-HOM condition (217 ms) were faster than in the 2L-IPSI condition (287 ms) [*p* = 0.001] and the latter were faster than in the 2L-DIAG condition (535 ms) [*p*<0.001].

#### Inhibition

**Figure 6:**
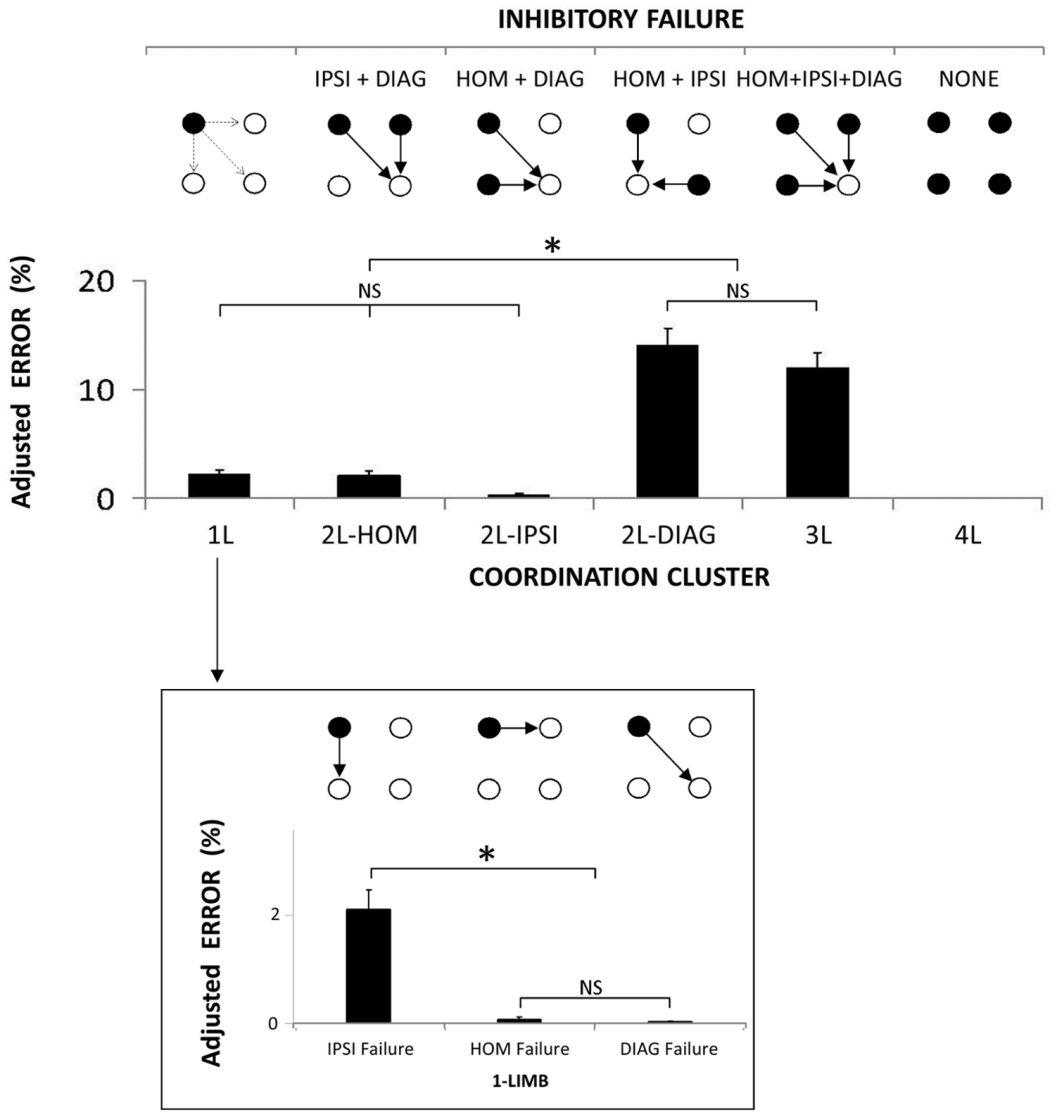
Decoupling of effector-specific networks. Upper panel. Adjusted error rates as a function of the six coordination clusters. Adjusted error rates of each coordination cluster reflect a specific failure of inhibitory interactions. Each circle stands for one effector-specific brain network. Black-filled circles illustrate networks associated with moving effectors and white-filled circles represent non-moving effectors. Arrows depict inhibitory interactions between moving and non-moving effectors. As normalized error rates are adjusted to the number of potential error sites here, we illustrated inhibitory interaction toward a single non-moving limb only. **Lower panel.** Adjusted error rates as a function of the three possible inhibitory failures in 1-limb reaction times. (Mean ± SEM; L = limb; DIAG = diagonal; IPSI = ipsilateral; HOM = homologous; NS = non-significant difference; * = significant difference).

For *adjusted error rates*, the 1-way (Coordination Cluster) ANOVA demonstrated a significant main effect [F(4, 140) = 62.06; *corr. p*<0.001; η^2^_P_ = 0.639] (Figure 6, upper panel). Post-hoc tests revealed that adjusted error rates in the 3L and 2L-DIAG conditions were not different from each other (12 vs. 14%, respectively) [*p* = 0.420] but they were higher than in the remaining coordination clusters [all *p*<0.001]. Error rates in the 1L (2%), 2L-HOM (2%) and 2L-IPSI (<1%) conditions were not different from each other [all *p*>0.377].

The 1-way (1 Limb) ANOVA demonstrated a significant main effect [F(2, 70) = 34.55; *corr. p*<0.001; η^2^_P_ = 0.497] (Figure 6, lower panel). Post-hoc tests revealed a higher adjusted-error rate in the ipsilateral limb (2%) compared to the homologous and diagonal limbs (both <1%) [both *p*<0.001] which were not different from each other [*p* = 0.985].

#### Processing complexity for the control of limb movements

Here, we report results that aim at revealing the effects of limb and laterality (1); establishing ordinal scales of recruitment (2) and selection complexity (3); and providing a ratio scale of the actual performance (4) that serves as a test of our ordinal approach of processing complexity.

### Recruitment and Selection Complexity Associated with Upper vs. Lower and Right vs. Left Limbs

For *simple RTs* which were assumed to be indicative of recruitment complexity, the 2×2×2 (Session × Limb × Laterality) ANOVA demonstrated a significant 3-way interaction [*F*(1, 35) = 4.60; *p* = 0.039; η^2^_P_ = 0.116]. Post-hoc tests revealed no practice effect from session 1 to 2 (between-mean-difference range = 3–4 ms) [all *p*>0.919]. Upper limbs were always faster than lower limbs when compared on a given session (between-mean-difference range = 12–24 ms) [all *p*<0.048]. The effect of laterality was never significant (between-mean-difference range = 4–8 ms) [all *p*>0.057].

**Figure 7:**
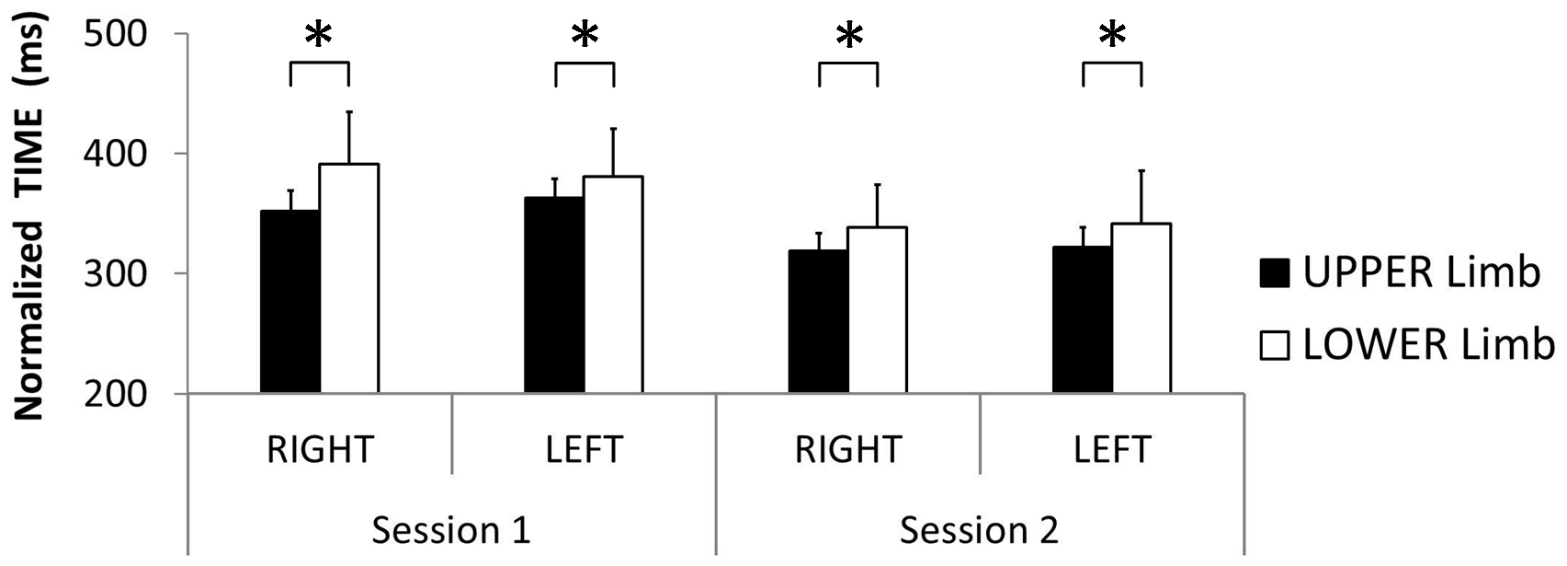
Processing complexity associated with upper vs. lower and right vs. left limbs. Normalized time performance in upper (black) and lower (white) limbs as a function of body side (right, left) and session (1, 2). (Mean ± SEM; * = significant difference).

For *normalized RTs* which were assumed to be indicative of selection complexity, the 2×2×2 (Session × Limb × Laterality) ANOVA demonstrated a significant 3-way interaction [*F*(1, 35) = 5.75; *p* = 0.022; η^2^_P_ = 0.141] (Figure 7). Post-hoc tests revealed a practice effect from session 1 to 2 for all limbs (between-mean-difference range = 32–53 ms) [all *p*<0.001]. Upper limbs were always faster than lower limbs when compared on a given session (between-mean-difference range = 16–39 ms) [all *p*<0.026]. The effect of laterality was never significant (between-mean-difference range = 3–11 ms) [all *p*>0.313].

For *normalized error rates*, the 2×2×2 ANOVA (Session × Limb × Laterality) revealed that neither the 3-way interaction [*F*(1, 35) = 0.27; *p* = 0.604; η^2^_P_ = 0.008], nor the 2-way Session × Limb [*F*(1, 35) = 0.14; *p* = 0.715; η^2^_P_ = 0.004], Session × Laterality [*F*(1, 35) = 0.48; *p* = 0.494; η^2^_P_ = 0.013] and Limb × Laterality [*F*(1, 35) = 0.01; *p* = 0.918; η^2^_P_<0.001] interactions, nor the Session [*F*(1, 35) = 3.56; *p* = 0.067; η^2^_P_ = 0.092], Limb [*F*(1, 35) = 1.82; *p* = 0.186; η^2^_P_ = 0.049] and Laterality [*F*(1, 35) = 1.80; *p* = 0.188; η^2^_P_ = 0.049] main effects reached significance.

### Recruitment Complexity of Coordination Clusters

**Figure 8:**
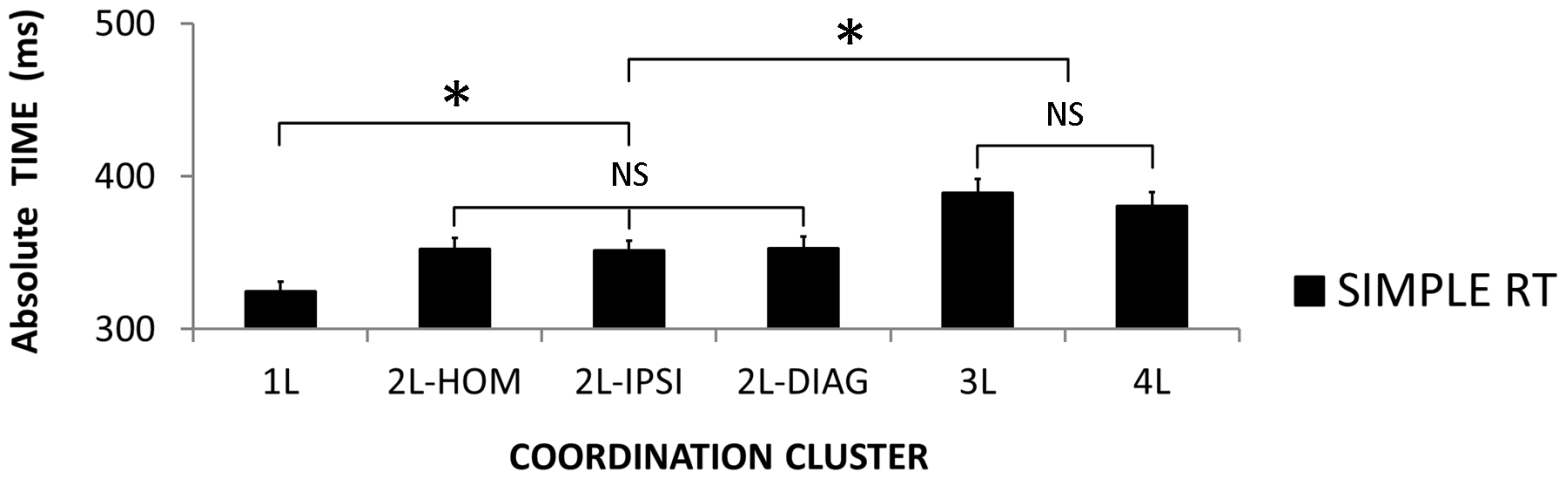
Recruitment complexity of coordination clusters. Absolute time in the simple RT condition as a function of the six coordination clusters. (mean ± SEM; L = limb; DIAG = diagonal; IPSI = ipsilateral; HOM = homologous; NS = non-significant difference; * = significant difference).

For *simple RTs*, the 2×6 (Session × Coordination Cluster) ANOVA revealed a main effect of Coordination Cluster [*F*(3, 175) = 25.56; *corr*. *p*<0.001; η^2^_P_ = 0.422] (Figure 8) but no significant 2-way interaction [*F*(5, 175) = 1.31; *corr*. *p* = 0.273; η^2^_P_ = 0.036] nor Session main effect [*F*(3, 35) = 0.57; *p* = 0.453; η^2^_P_ = 0.016]. Post-hoc tests revealed that simple RT in cluster 1L (325 ms) was faster compared to clusters 2L (352 ms for the three of them) [*p*<0.001] which were faster than clusters 3L (388 ms) [all *p*<0.001] and 4L (381 ms) which were not different from each other [*p* = 0.735].

### Selection Complexity of Coordination Clusters

**Figure 9:**
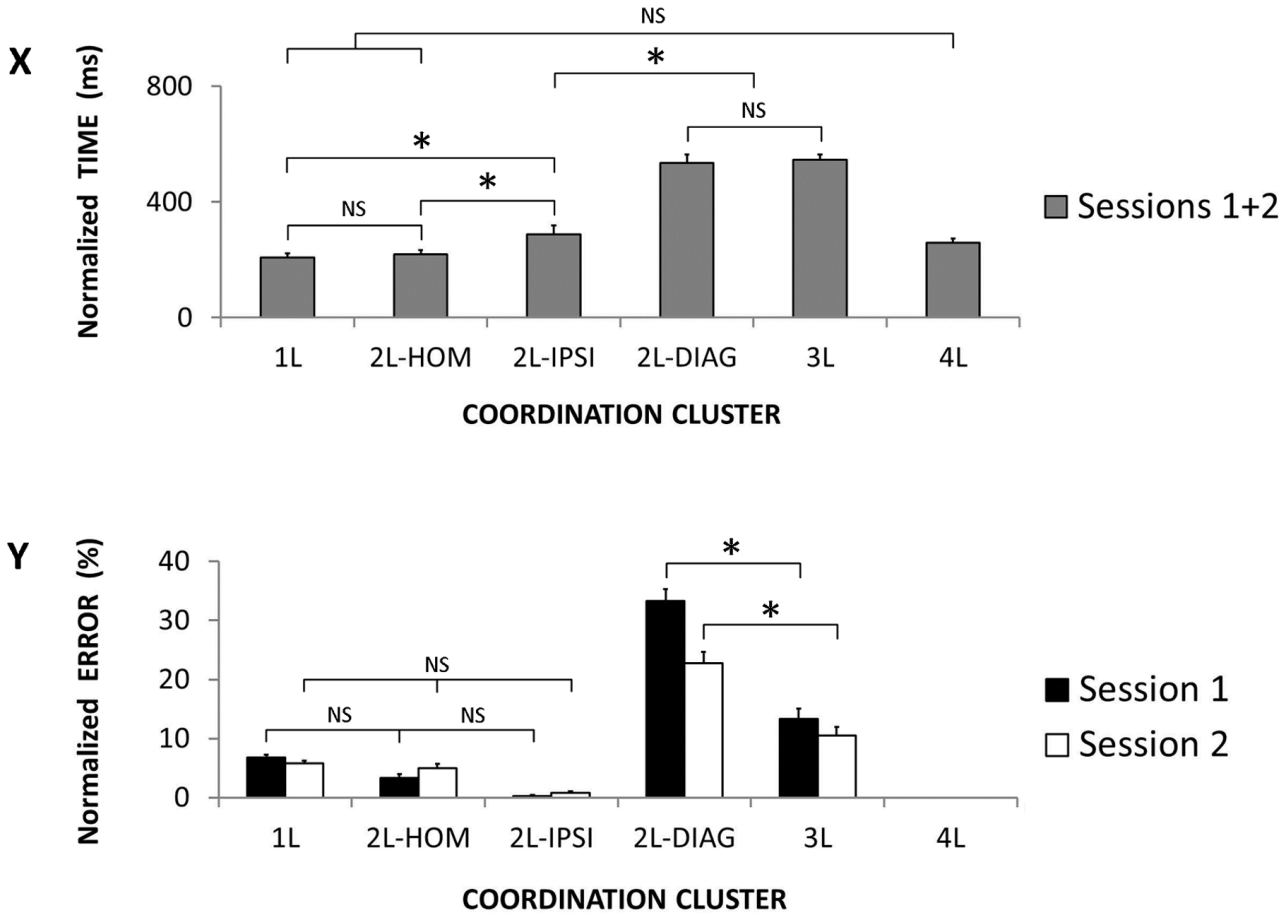
Selection complexity of coordination clusters. **X.** Normalized time performance (gray columns) as a function of the six coordinations clusters. **Y.** Normalized error rate as a function of the six coordination clusters in Session 1 (black columns) and 2 (white columns). (Mean ± SEM; L = limb; DIAG = diagonal; IPSI = ipsilateral; HOM = homologous; NS = non-significant difference; * = significant difference).

For *normalized RTs*, the 2×6 (Session × Coordination Cluster) ANOVA did not reveal a significant interaction effect [*F*(5, 175) = 2.08; *corr*. *p* = 0.097; η^2^_P_ = 0.056], whereas significant Session [*F*(1, 35) = 20.95; *p*<0.001; η^2^_P_ = 0.374] and Coordination Cluster [F(5, 175) = 140.83; *corr. p*<0.001; η^2^_P_ = 0.800] main effects were observed. The main effect of session indicated a practice-induced time reduction from session 1 to 2 (360 vs. 325 ms). Regarding the main effect of coordination cluster (Figure 9X), latencies required for clusters 2L-DIAG and 3L were not significantly different from each other [*p* = 0.999] (535 vs. 544 ms) and were longer than all the other clusters [all *p*<0.001]. Conversely, the time required for performing clusters 1L, 2L-HOM and 4L were not significantly different from each other (208, 218 and 258 ms, respectively) [all *p*>0.082] and mainly shorter than the other clusters. The only exception was the absence of a significant difference between clusters 2L-IPSI (287 ms) and 4L [*p* = 0.618]. In sum, latencies for coordination clusters 2L-DIAG and 3L were the longest, 1L, 2L-HOM and 4L were the shortest, and 2L-IPSI was somewhat positioned in between.

For *normalized error rates*, the 2×5 (Session × Coordination Cluster) ANOVA demonstrated a significant interaction [*F*(4, 140) = 5.71; *corr*. *p* = 0.003; η^2^_P_ = 0.140] (Figure 9Y). Post-hoc tests revealed a significant effect of practice from session 1 to 2 for coordination cluster 2L-DIAG only (33 vs. 23% errors) [*p*<0.001]. The error rate in coordination clusters 2L-DIAG was the highest in both sessions [all *p*<0.001]. In session 1, the error rate in coordination cluster 3L (13%) was the second highest [all *p*<0.014]. Error rate in 1L (7%) was significantly higher than in 2L-IPSI (<1%) [*p*<0.009]. Error rate in 2L-HOM (3%) was not different from 1L and 2L-IPSI [both *p*>0.617]. In Session 2, error rate in coordination cluster 3L (11%) was larger than in coordination clusters 2L-IPSI (1%) [*p*<0.001] but not different from 1L (6%) [*p* = 0.207] and 2L-HOM (5%) [*p* = 0.052]. The error rates in coordination cluster 1L, 2L-HOM and 2L-IPSI were not different from each other [all *p*>0.117]. To sum up, error rate for coordination cluster 2L-DIAG was the highest, 3L was at an intermediate error rate, and 1L, 2L-HOM and 2L-IPSI globally showed the lowest error rate.

### Absolute Performance in Simple and Choice MUL-RT

**Figure 10:**
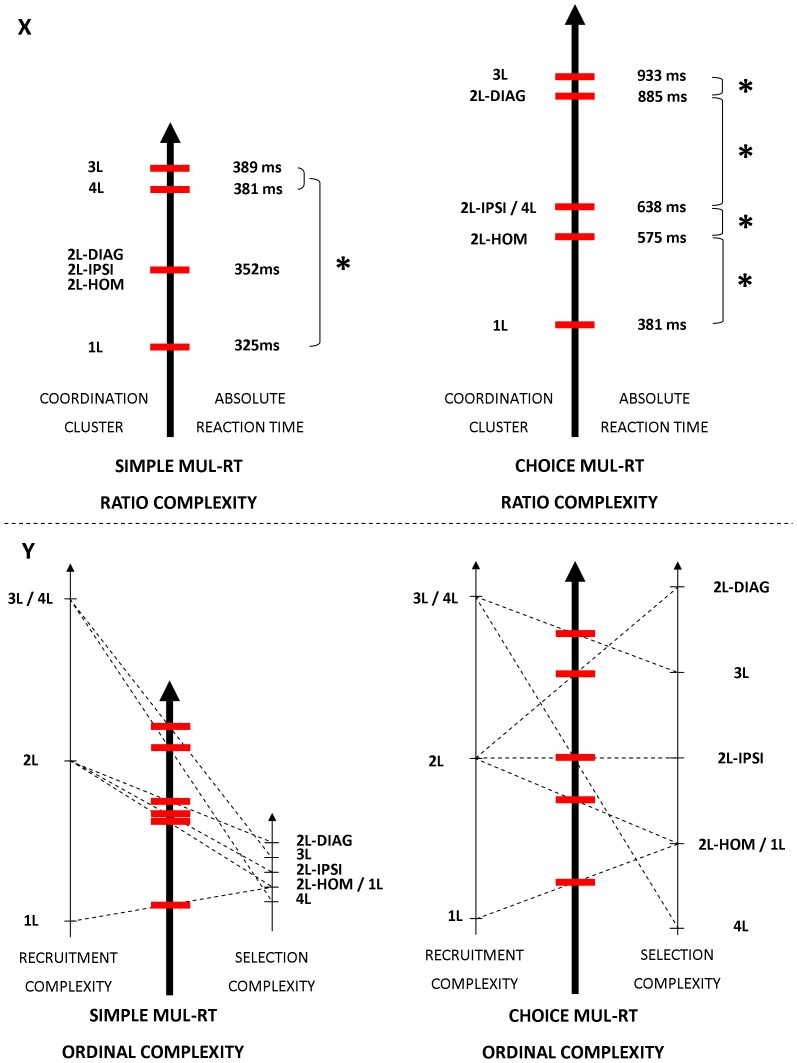
Weighting of the recruitment and selection principles accounts for overall complexity. **X. Ratio complexity.** Overall ratio complexity of the different coordination clusters in simple (left-hand panel) and choice (right-hand panel) MUL-RT is estimated based on the absolute time-duration measures. **Y. Overall processing complexity is extrapolated, based on the ordinal scales of recruitment and selection complexity.** Overall ordinal complexity (middle bold arrow) is extrapolated from the association of the number (left regular arrow) (3L = 4L>2L>1L) and interaction (right regular arrow) (2L-DIAG>3L>2L-IPSI>1L = 2L-HOM>4L) ordinal complexity. The arrows indicate the direction of increased complexity. Length of the arrows represents the relative contribution (weight) of each principle to the overall complexity of the task. This relative contribution determines the order of the coordination clusters’ overall complexity as indicated by red horizontal bars where dotted lines cross the bold arrow. In simple RTs, the recruitment complexity is more heavily weighted than selection complexity. As a consequence, overall ordinal complexity in the simple RT condition follows the pattern of recruitment complexity. From simple to choice MUL-RT, the contribution/weighting of selection complexity increases relative to recruitment complexity (increased length of the selection complexity but not recruitment complexity arrow). As a result overall complexity in the choice MUL-RT (3L>2L-DIAG>4L = 2L-IPSI>2L-HOM>1L) is no longer solely governed by the recruitment principle but reflects a similar contribution from the recruitment and selection principles (similar length of the arrows). Extrapolated complexity of the different coordination clusters matches the ones observed in the ratio scale for both the simple and choice MUL-RT. Therefore, overall complexity of a given coordination cluster in a given RT condition can be explained by a weighted combination of the recruitment and selection principles. (L = limb; DIAG = diagonal; IPSI = ipsilateral; HOM = homologous; NS = non-significant difference; * = significant difference).

For *absolute RTs* (simple and choice RTs), the three-way interaction of the 2×2×6 (Session × Block × Coordination Cluster) ANOVA did not reach significance [F(5, 175) = 2.08; *corr. p = *0.097; η^2^_P_ = 0.056] whereas the three lower order Session × Block [F(1, 35) = 20.95; *p*<0.001; η^2^_P_ = 0.374], Session × Coordination Cluster [F(5, 175) = 4.11; *corr. p = *0.006; η^2^_P_ = 0.105] and Block × Coordination Cluster [F(5, 175) = 139.83; *corr. p*<0.001; η^2^_P_ = 0.800] interactions were observed. As confirmed by post-hoc tests, the Session × Block interaction indicated an effect of practice between session 1 and 2 in choice RT (719 vs. 681 ms) [*p*<0.001] but not simple RT condition (359 vs. 356 ms) [*p* = 0.893]. The Session × Coordination Cluster interaction demonstrated a practice effect between Session 1 and 2 in coordination clusters 2L-DIAG (632 vs. 601 ms) and 3L (685 vs. 637 ms) [both *p*<0.005]. Post-hoc analysis of the Block × Coordination Cluster interaction revealed that all coordination clusters were longer in the choice RT as compared to the simple RT condition (between-mean-difference range = 208–608 ms) [all *p*<0.001]. In the simple RT condition, none of the differences among coordination clusters reached significance [all *p*>0.085] except for the ones between coordination cluster 1L (325 ms) and coordination clusters 3L (389 ms) and 4L (381 ms) [both p<0.002] (Figure 10X, left-hand panel). In the choice RT condition, coordination cluster 3L (933 ms) was significantly longer than coordination cluster 2L-DIAG (885 ms) [*p = *0.014], which was significantly longer than coordination clusters 2L-IPSI (638 ms) and 4L (638 ms) [both p<0.001] which were not significantly different from each other [p>0.999] and longer than coordination cluster 2L-HOM (575 ms) [*p*<0.001] which was longer than 1L movements (381 ms) [*p*<0.001] (Figure 10X, right-hand panel).

Overall, results of the absolute data clearly revealed two patterns of time performance with (a) shorter latencies in the simple-RT condition that were primarily determined by the number of limbs involved (recruitment principle), and (b) longer and more coordination-cluster-specific durations in the choice RT condition (selection principle).

## Discussion

Here, we used a multilimb RT task to first investigate the mechanisms of limb selection. Results supported our model of coupling/decoupling interactions (Figure 2). Second, we studied the determinants of processing complexity for the control of limb movements. Specifically, we tested (1) the effect of limb and body side on recruitment and selection complexity while disregarding the coordination clusters, (2) the effect of the number of effector-specific networks to be recruited, (3) the effect of the nature of the coupling/decoupling interactions involved and (4) whether weighting of the recruitment and selection complexity/principle could determine performance differences across coordination clusters in both the simple and choice MUL-RT. Results did not reveal performance differences between right versus left limbs but moving upper limbs required less processing time than moving lower limbs. RT performance in the different coordination clusters was dependent on a weighted combination of the recruitment and selection principle.

### The Model of Coupling/Decoupling Interactions

To test our model of coupling/decoupling interactions, we investigated excitatory and inhibitory interactions between homologous, ipsilateral and diagonal limb movements. To this aim, normalized RT (choice minus simple RT) required to select two effector-specific networks was considered to be indicative of the level of excitatory interaction between these networks (coupling) and adjusted error rate was considered to be indicative of the failure of the inhibitory interaction (decoupling).

The shortest normalized time was observed when two homologous limbs were recruited (Figure 5) and suggested high excitatory interaction within this effector couple, as reported in the literature [10], [11], [12], [13], [14], [15], [16], [17], [18], [19], [20], [21], [22], [23], [24], [25], [26], [27]. The low rate of homologous errors in the 1L condition (Figure 6, lower panel) was also consistent with existing literature [34], [35], [36], [37] and suggested that the excitatory homologous interaction was efficiently inhibited [10], [11], [43]. These results supported the excitatory/inhibitory homologous interaction (++++/−−−−) proposed in our model (Figure 2).

The longest normalized time was observed when two diagonal limbs were recruited (Figure 5) which was interpreted as low excitatory interaction within this couple of limbs. The low rate of diagonal errors in the 1L condition (Figure 6, lower panel) was interpreted to suggest that this excitatory interaction was efficiently inhibited. These results support the nature of the diagonal interaction (+/−) proposed in our model that is weaker than the homologous interaction (Figure 2). In terms of inhibition, the 1L condition mainly reflected an ipsilateral failure of inhibition (Figure 6, lower panel) [34], [35], [36], [37]. Adding the possibility for a diagonal failure of inhibition (2L-HOM) to the 1L condition had no effect on the error rate (Figure 6, upper panel) supporting the idea of an overall (i.e., both excitatory and inhibitory) weakness of the diagonal interaction [8], [9]. Similarly, the addition of the possibility for diagonal failure of inhibition (3L) to the homologous and ipsilateral possibilities (2L-DIAG) did not increase the error rate either (Figure 6, upper panel).

When two ipsilateral limbs were recruited, the normalized time was intermediate (Figure 5). The high rate of ipsilateral errors in the 1L condition supported the literature [34], [35], [36], [37] and suggested that the degree of inhibitory interaction was not always sufficient to overcome the intermediate level of excitatory interaction (Figure 6, lower panel). These results supported the imbalance between excitation and inhibition of the ipsilateral interaction (+++/−−), as proposed in our model (Figure 2).

In sum, our results appeared consistent with the proposed model of coupling/decoupling interactions (Figure 2).

### Recruitment and Selection Complexity for Right vs. Left and Upper vs. Lower Limb

For the assessment of processing complexity *simple RT* was considered to be an indicator of the recruitment complexity while *normalized data* (choice minus simple RT) was considered an indicator of the selection complexity (please see Methods).

*Simple and normalized* RTs were shorter for upper relative to lower limbs, whereas right and left limb values were similar (Figure 7). These results are consistent with previous studies that investigated simple RT differences among the four limbs [48], [53], [54], [55]. Faster *simple* RTs in upper relative to lower limbs could be explained by lower nerve conduction velocities and longer nerve pathways for feet relative to hands [56], [57]. In addition, a musculoskeletal/biomechanical account for this upper versus lower limb difference is also viable as it may take more time to overcome the inertia of the foot relative to the hand due to the higher mass of the foot. However, our normalized results called for an additional account for upper- vs. lower-limb differences. This difference possibly suggests a higher level of complexity for selecting lower- relative to upper-limb movements. This difference may be related to the daily use of complex hand/arm movements for reaching and grasping which may have improved the efficiency of processing in upper limbs relative to lower limbs. Conversely, the absence of normalized RT differences between right and left limbs suggested that processing complexity was not body-side dependant.

The absence of differences in normalized error rates among the four limbs suggested that the time variable was more sensitive than the error variable in revealing a difference of complexity between the processing of upper versus lower limbs.

### How Recruitment and Selection Principles Contribute to overall Processing Complexity for Different Coordination Patterns

Based on our results, we attempted to define ordinal scales of recruitment and selection complexity (Figure 10X). Results showed maximum recruitment complexity in coordination clusters 3L and 4L, minimum recruitment complexity in the 1L, and intermediate recruitment complexity for the three 2L coordination clusters (homologous, ispsilateral and diagonal) (Figure 8). To sum up, the ordinal scale of recruitment complexity was the following: 3L = 4L>2L>1L.

With regard to selection complexity (Figure 9), normalized RTs were the longest for 2L-DIAG and 3L conditions which were not different from each other, but, error rate was higher in 2L-DIAG. The difficulty with performing 2L-DIAG as compared to the remaining 2L conditions may primarily be due to the need for inhibiting both strong (homologous) and intermediate (ipsilateral) excitatory interactions (Figure 6, upper panel) with respect to the two non-moving limbs. The higher complexity observed in the 2L-DIAG compared to 3L could possibly be explained by the necessity to inhibit two limbs from ipsilateral and homologous excitatory interactions in the former and only one limb in the latter. Overall, error rates in conditions 1L, 2L-HOM and 2L-IPSI were the lowest. However, RT in 2L-IPSI was longer compared to 1L and 2L-HOM. The higher complexity associated with ipsilateral compared to homologous limb recruitment could be explained by reduced excitatory interactions within ipsilateral limbs (Figure 5). The low complexity exhibited in 1L could be due to the absence of double/triple excitation towards a given non-moving lilmb (Figure 6, lower panel). The 4L condition was considered the least complex coordination cluster as it complied with only excitatory (lowest level) and no inhibitory interactions. In other words, when considering both normalized RTs and normalized error rates as indicators of selection complexity, the following ordinal scale emerged: 2L-DIAG>3L>2L-IPSI>1L = 2L-HOM>4L.

Based on these two ordinal scales, we extrapolated the overall ordinal complexity of the simple and choice MUL-RT (Figure 10Y). This approach was efficient in reproducing the absolute results we obtained on a ratio scale (ms) (Figure 10X). Complexity in the simple RT condition was clearly governed by the recruitment principle with little contribution of the selection principle. Conversely, the recruitment principle did not appear to be sufficient to fully explain the choice RT differences across coordination modes. Indeed, the 4L condition did not exhibit the longest RT and the three 2L conditions showed different RTs. Specifically, absolute choice RTs were gradually decreasing as follows: 3L>2L-DIAG>2L-IPSI = 4L>2L-HOM>1L. Taken together, these results suggested that, relative to the recruitment principle, the selection principle had a stronger weighting in choice compared to simple RT conditions (Figure 10). Overall, we demonstrated that each coordination pattern is exposed to both the number and selection principles which ultimately determine the complexity of the task (Figure 10Y). Additionally, the results showed that the relative weighting of these principles depends on the nature of the RT (simple vs. choice).

### Effects of Practice

Overall, the observed differences among conditions were robust against training effects even though general improvement occurred with practice. Results failed to reveal an effect of practice for simple RTs, whereas such an effect was demonstrated for choice RTs. This simple- vs. choice-RT difference resulted in a main effect of practice for the normalized time, suggesting that selection complexity but not recruitment complexity can be decreased with practice. Analysis of coordination clusters revealed that this practice effect was mainly due to improved RTs in conditions 2L-DIAG and 3L. In terms of errors, a significant practice effect was only evident in 2L-DIAG. In sum, there was a clear practice effect for the most complex conditions (3L and 2L-DIAG).

## Conclusion

Selection complexity at the level of limb couples was accounted for by a model of coupling/decoupling interactions (Figure 2). The behavioral evidence for this model is strong and consistent with both existing literature and our current dataset (please see Introduction and Results, respectively) but the neural evidence is still incomplete at best. Therefore, additional studies including neurophysiological and imaging techniques are warranted to evaluate the anatomical and functional connectivity responsible for the level of complexity associated with the selection of limb-specific sensorimotor networks.

Our ordinal approach of processing complexity (Figure 10) suggested that in choice RT, selection- and recruitment-complexity contributions to the overall complexity are similar.
